# Ethnozoological study of medicinal animals used by the inhabitants of the Kucha District, Gamo Zone, Southern Ethiopia

**DOI:** 10.1186/s13002-024-00714-8

**Published:** 2024-08-02

**Authors:** Meselech Mengistu, Mulugeta Kebebew, Victor Benno Meyer-Rochow

**Affiliations:** 1 Department of Biology, College of Natural and Computational Sciences, Arba Minch University, 4400 Arba Minch Zuria, Ethiopia; 2 Department of Ecology and Genetics, University of Oulu, 90014 Oulu, Finland

**Keywords:** Medicinal animals, Fidelity level, Informant consensus factor, Relative frequency of citation, Ethnozoology, Indigenous knowledge, Traditional remedies

## Abstract

**Background:**

An ethnozoological study of medicinal animals in the Kucha district, Gamo zone, Southern Ethiopia, was conducted to investigate and document the use of traditional medicinal animals and the associated indigenous knowledge. Tribal people still make abundant use of animals and their parts to manage diseases in humans and even livestock.

**Method:**

A cross sectional study design and purposively sampling techniques were used. Data were collected from 132 respondents based on semi structured questionnaires. Focus group discussions (FGD) and Key informant interviews (KII) were conducted; Fidelity level (FL), Relative frequency of citation (RFC) and Informants’ consensus factor (ICF) were used to analyze species preference and importance.

**Results:**

A total of 24 medicinal animals were identified with 13 species (54.2%) being mammals of which 5 species (20.8%) dominated. They were followed by arthropods, reptiles and fishes. Seven out of the total were domestic species (29%) and 17 (70%) were wild animals. The majority of these animals, i.e. 22 (91.7%), were used to treat human ailments; whereas 2 (8.3%) were used to treat livestock ailments. The ICF values varied from 0.8 to 1.The highest FL value (98%) was linked to the cow (cattle), the lowest (1.5%) to the scorpion. The RFC value (1.0) was highest for the cow and lowest (0.02) for the scorpion. Honey, milk, and butter were the most commonly used therapeutic animal products, but regarding direct uses, fresh/raw meat dominated. Out of ten ailment categories, headaches had the lowest ICF value (0.8). All others scored at least 0.9.

**Conclusions:**

In rural areas, sick people often do not only have limited access to modern medical facilities, they actually prefer traditional treatments considering them to be more reliable and effective. It is therefore important to focus on documenting, conserving, and safeguarding the indigenous knowledge so that strategies to manage the traditional wisdom can be implemented in the future*.* To achieve these goals, it is important to make sure that medicinal animal species are available in sufficient numbers and neither threatened by habitat changes or overexploitation.

## Introduction

One aim of ethno-medicine is to record and document the indigenous knowledge held by local people on the various therapeutic uses of minerals, flora and fauna. This includes obtaining an inventory of medicinally useful animals, identifying and recording the animals’ local names and taking notes of the species’ cultural importance and appreciation [1, 2]. Animals and their products, used therapeutically in different countries, cultures and societies, are an important part of these studies. The traditional medicinal knowledge of indigenous people across the globe has played a significant role in identifying species which are endowed with bio-medically active compounds, effective in treating a variety of health conditions [3–5]. From the earliest days of recorded history animals and their products have been used in preparations of traditional remedies in various cultures [6].

When healing practices involve herbal medicines, spirituality, exercises and manual manipulations to diagnose, treat or prevent an ailment or illness, this is known as folk or traditional medicine [7]. Healing with animal-derived medicines constitutes a major alternative among other known therapeutic practices in the world [8–14]. Mammals, birds, reptiles, fish, arthropods and other invertebrates as well as their products such as meat/fat, liver, bile, skin, horn, bone, tusks and teeth, hooves, feathers, blood, saliva, feces, venom, shells, eggs, nest material, etc., are part of the armamentarium of traditional healers the world over and serve as important medicinal ingredients [3–5, 9, 13–17].

Animals not only contribute to traditional but also to modern medicine, with natural extracts being used by pharmaceutical companies as raw material for the manufacture of a variety of drugs. For instance, of the 252 chemicals selected as essential by the World Health Organization (WHO), 11.1% are derived from plants and 8.7% from animals [18]. Additionally, of the 150 prescription drugs used in the United States of America (USA) in the year 2000**,** 27 had an animal origin [1, 19].

In Ethiopia different ethnic groups and tribal people use various animals and their products for treating human ailments. An ethnozoological survey of traditional medicinal animals used by the people of the Kafta Humera district identified 16 medicinal animal species for treatments of 18 different human disorders [20]. A study in the Degu'a Tembien region of Northern Ethiopia identified 23 animal species and their products to be therapeutically important [21]. In the Amaro district of southern Ethiopia over 21 medicinal animals were recorded as part of the traditional health care by the Kore people [22], and in the Metema district of the Semien Gondar Zone of the Amhara Region, 51 locally available medicinal animal species were used for treating at least 36 kinds of ailments [15]. In the Arba Minch Zuriya district of the Gamo Zone in southern Ethiopia only 20 species and/or their products were found to be commonly used therapeutically in combating diseases and disorders [13].

The aim of our research was twofold. In Ethiopia most ethnobiological studies have focused on the traditional knowledge of plants and much less on animals [23]. We are therefore trying to address the dearth of knowledge regarding medicinal animal species in the Kucha District, whose inhabitants have not earlier had a chance to explain which species of animals they appreciated as a source of material to fight diseases and other ailments with. The research focuses on identifying the medicinal animal species, the parts of animals used for medicinal purposes, the methods of preparation and routes of administration as well as types of illnesses and disorders that the local tribal people believe can be cured with animal-based treatments. As ‘western medicine’ is frequently seen as foreign and not locally developed, it is regarded with suspicion, not having stood the test of time. Moreover, it is usually more expensive, harder to obtain and administered by people that are less trusted than the local healers. Therefore, in order to make the locals understand that traditional and western/modern approaches to fight diseases are complementary, it is important to know why and how the locals use certain animals and their products in their traditional treatments. This also allows us to learn from the traditional practitioners and to find out what makes some folk medicines potent and appreciated by the local community.

## Materials and methods

### Description of the study area

**Fig. 1:**
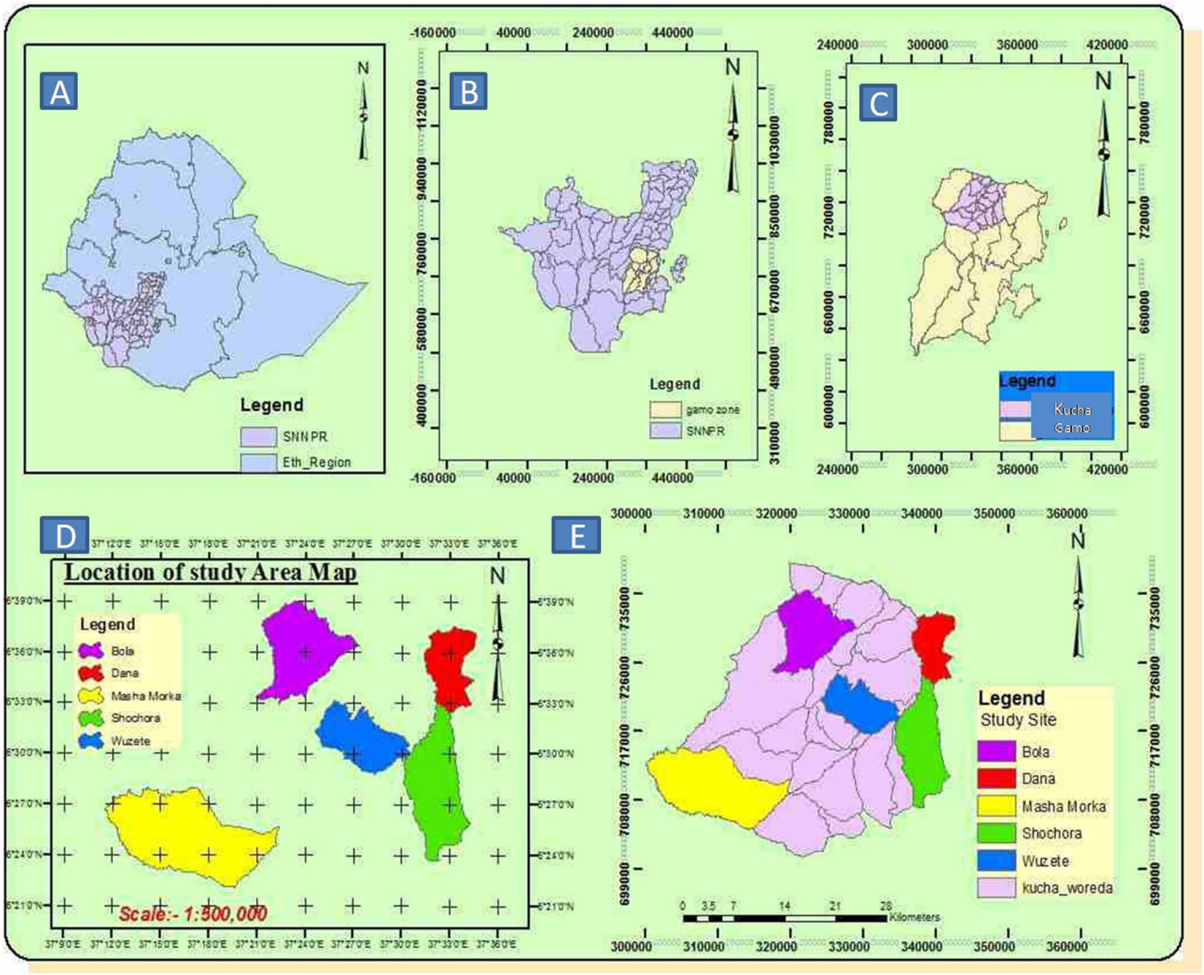
Map showing the study area **A** Ethiopia with SNNPR (Southern Nations, Nationalities, and Peoples’ Region) and GPS coordinates of 9.1450N and 40.4897E, **B** SNNPR with Gamo zone, **C** Gamo zone with Kucha district in blue, **D** Kucha district with study sub-districts (*kebeles*), **E** Study sub-districts (*kebeles*) within the Kucha district and GPS coordinates of 6.499998N and 37.333332E. Numbers on the x and y axes are distances in km

The study was conducted in the Kucha district, Gamo Zone, South Ethiopian National Regional State (Fig. 1A–E). It is located at 177 km from Arba Minch town, the zonal capital, and 390 km from Addis Ababa, capital of the country. Information on geographic features, climate details and population structures of the district are obtained from Tabofie et al. [24]. One distinguishes three agro-climatic zones and geographically the region’s distinct attributes can be explained as highland, mid-altitude and lowland. The altitude of the study area is between 900 and 2400 m above sea level. Rainfall is between 1100 and 1600 mm per annum and average temperature of the district is between 17.6 °C and 27.5 °C. The study area lies in the dry evergreen montane forest and the grass land complex in southern Ethiopia, containing different herbs, shrubs and trees. However, there are increasingly more encroachments into the forest area due to the pressure of an increasing population, but a natural forest as well as some planted regions which harbour a wide range of wildlife, are also still present. Most of the farmers in the area depend on agricultural activities for their livelihood. The Kucha district has a total population of 159,779 of which 78,292 (49.8%) are males and 81,487 are females. Information on the religions, ethnicities, and languages of the district is provided in Table 1.

**Table 1:** Proportion of inhabitants (%) in Kucha District by religion

Religions			
Protestand	Ethiopian orthodox	Catholic	Other beliefs
49.83	45.73	3.36	1.08
Ethnicities of the Kucha district			
Gamo (98.52)		Others 1.48	
Languages of the Kucha district			
Gamotho (99.01)		Amharic (0.99)	

Mixed agriculture and weaving are widely practiced among the Gamo people. Crop cultivation (teff, maize, sorghum, coffee, yam, cassava, mango, enset, sweet potato, taro, bananas, papaya, mango, and avocado) is the primary and most important agricultural activity of the community; while, livestock production (cattle, sheep, goats, and poultry) is the second most widely practiced agricultural activity.

### Reconnaissance and study site selection

A reconnaissance in the Kucha district was conducted from September 10–30, 2021 to select specific study sites. The study sites were purposively selected based on the recommendation from elders, local authorities, and knowledgeable persons, availability of traditional medicines and traditional practitioners. Out of 24 peasant associations the study was carried out in five peasant association areas, involving 3041 households (Table 2).

**Table 2:** Number of respondents from each Peasant Association visited

Peasant associations	Total households (*N*)	Respondents (*n*)
Bolla	720	31
Dana	348	15
Morka	522	23
Schchora	821	36
Wuzate	630	27
Total	3041	132

### Informant selection and data collection

A total of 132 individuals with ages of 25 years and above were selected from five peasant associations (Fig. 1) purposively based on their intensive knowledge of the medicinal animals. The informants were selected from the local people of the study area and were asked to share their general knowledge of medicinal animals. The selection of key informants when recording indigenous knowledge safeguarded by traditional healers is most important. Ultimately, 10 key informants (8 males and 2 female) and 36 focus group discussion participants (30 males and 6 female) became involved, based on the recommendation by elders and local authorities (Table 3). Local healers were also considered as key informants since they were expected to have intensive knowledge of medicinal animals as well as people living in their areas. Interviews and discussions were conducted in the Gamoto language as 99% of the local populations could understand the language and only 1% were Amharic speakers.

**Table 3:** Information regarding key informants and focus group discussion participants

Selected area	Sample respondents						Sample technique
	Key informants			Focus group discussion			
	M	F	T	M	F	T	
Bolla	2	_	2	6	1	7	Purposive
Dana	1	_	1	5	1	7	Purposive
Morka	1	_	1	5	1	7	Purposive
Shochora	3	1	4	8	2	8	Purposive
Wuzate	1	1	2	6	1	7	Purposive
Total	8	2	10	30	6	36	

Ethnozoological data were collected from September to February 2021. Key informants shared their knowledge on the methods of preparation and modes of application of different medicinal animals that are used to treat both human and livestock ailments. Uses other than the medicinal ones, such as spiritual and magical ones of the mentioned animals were not neglected and also recorded.

Semi-structured interviews were conducted with 132 informants in the local language to collect ethnozoological data. The collected data contained information on the informants' names, sex, ages, and addresses; they listed the most common human and livestock ailments in the area, the local vernacular names of the medicinal animals, the medicinal parts/products of the animals, held information on whether humans or livestock (human/livestock) had their ailments treated, contained information on preparation methods of the traditional animal-based medicines and on the various modes of application/administration of the traditional medicines, and inquired about conservation and preservation issues, threats of medicinal animals and additional non-medical uses of the animals.

**Fig. 2:**
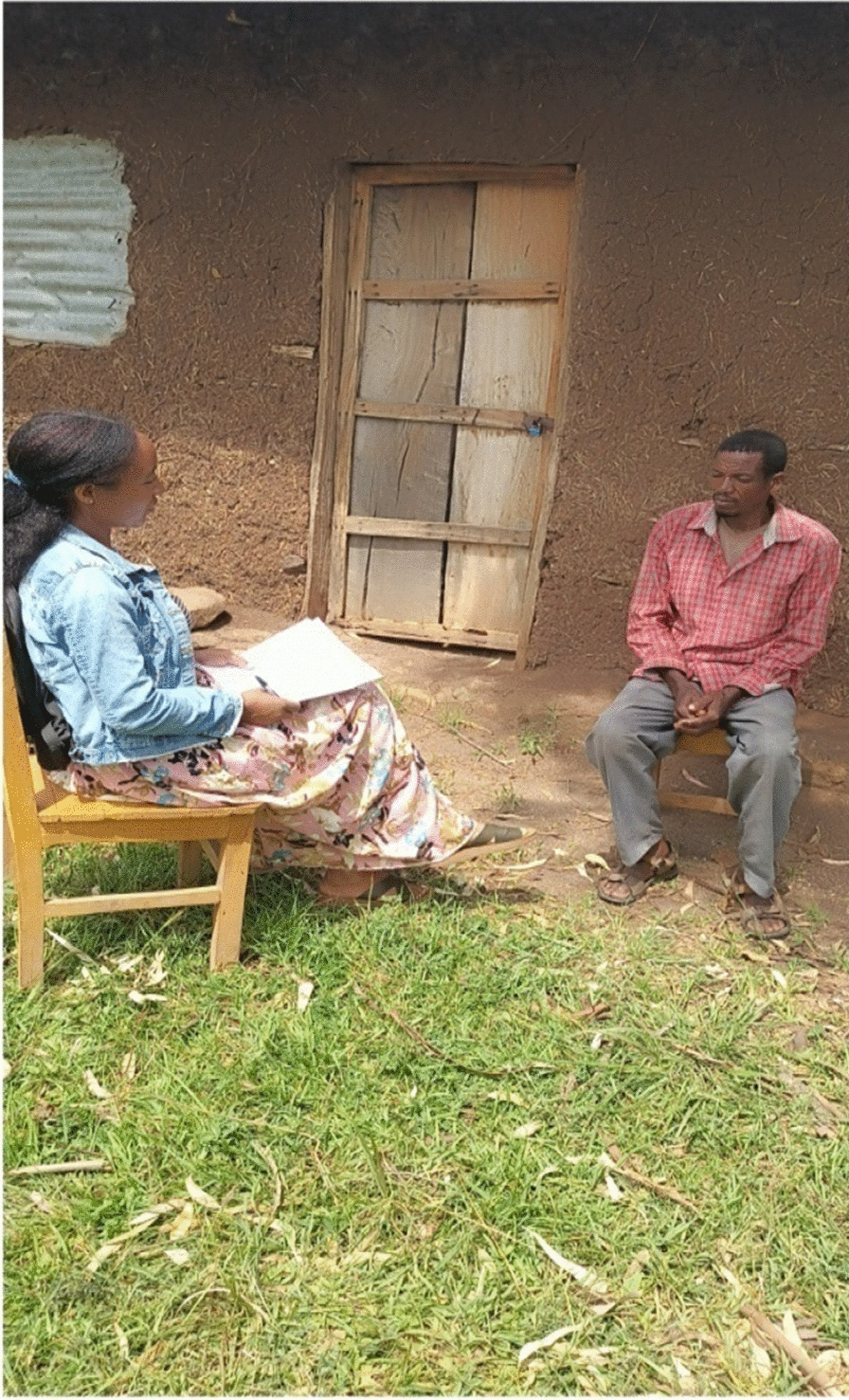
Investigator (left) with male key informant

**Fig. 3:**
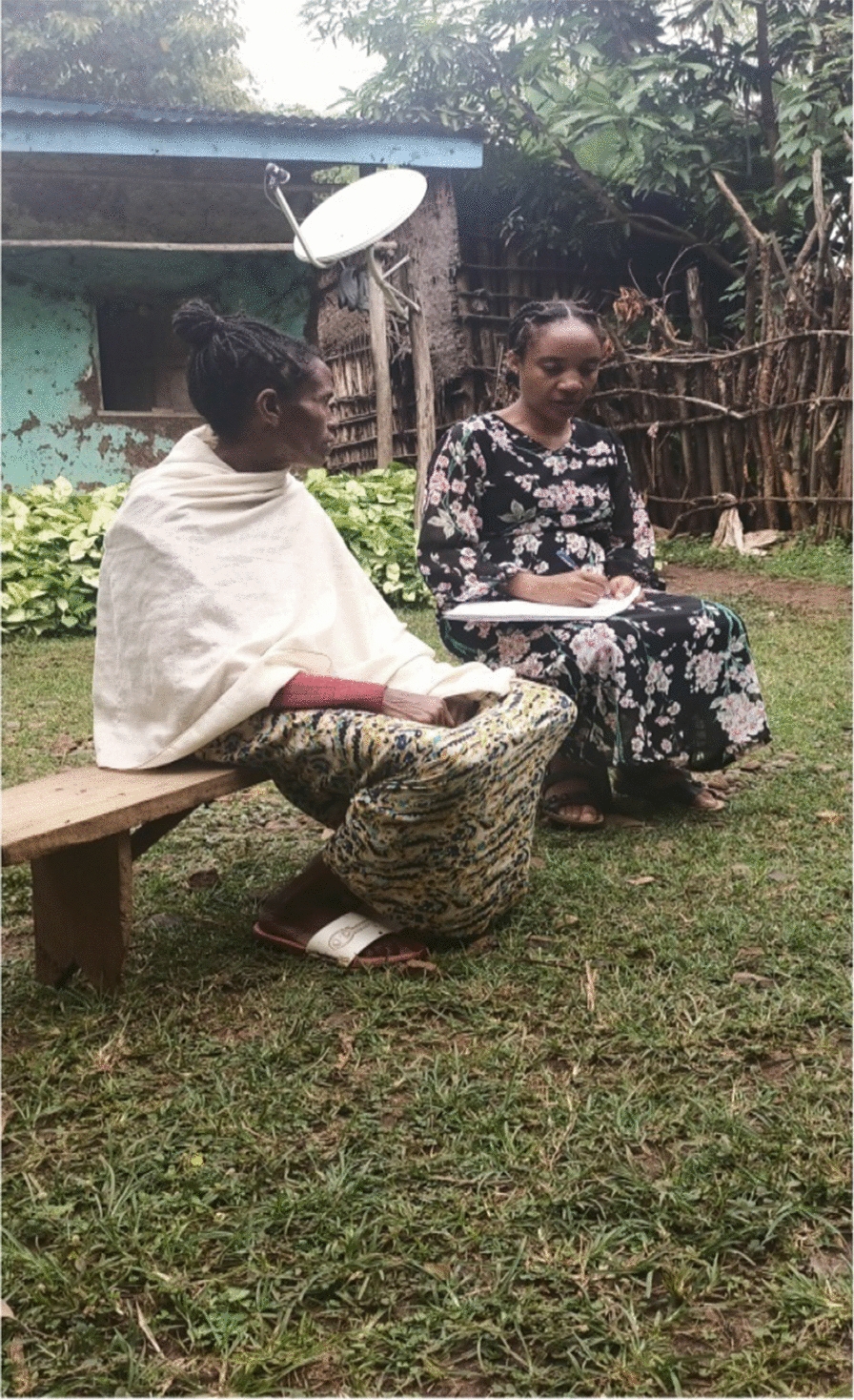
Investigator (right) with a female key informant

**Fig. 4:**
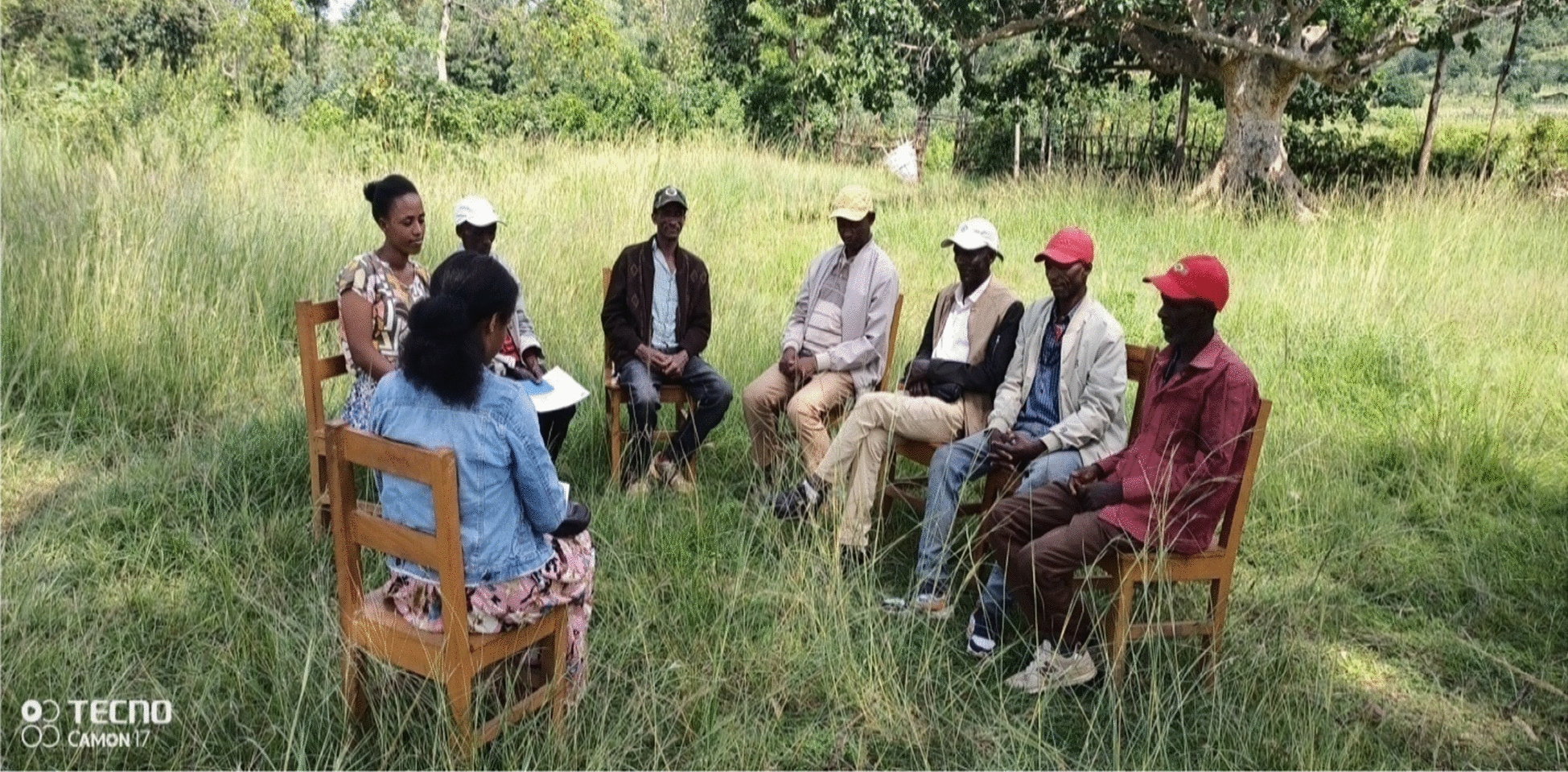
Researcher involved in group discussion with informants

Group discussions were held with informants at each peasant association before the ethnozoological data were collected (Figs. 2, 3) A typical discussion was based on a predetermined set of questions which were prepared by the investigator. The number of participants in each group was purposively determined by the investigator. Totaling 36 Focus Group Discussion with 30 males and 6 females, two females from Shochora and one female each from other peasant association participated in the Focus Group Discussion (Table 3). Participants in key informants and focus group discussion, based on their willingness, also participated in the interview-based questionnaires; the results of the interviews were obtained from concerned local elders, household heads and each Peasant association administrator. Discussions were held with each group at different times convenient to the participants of the group. During the discussions, an attempt was made to let the participants understand that their traditional knowledge and the continued practice of their art of traditional medicinal interventions would not be interfered with by the researcher (Fig. 4).

### Ethics declaration

Data collection commenced after permission was obtained from the Administrative Office of the Arba Minch University and the individuals who were willing to participate in the research had given their consent to be interviewed. Special ethical consideration was taken from the beginning to the end of the data collection. In view of the ethical considerations, approaching the informants was very systematic. Informants were told that the objective of the research was to compile and document the significance of the medicinal animals of the study area, but that this was not for a commercial purpose. This was substantiated and confirmed by showing letters from the administrative office of the district. Consequently, once the informants had understood and accepted the idea, they would freely give information on the medicinal species of animals in the area and their knowledge about the various species.

### Reliability of information

During the course of the study, each informant was visited two times within an interval of a few days in order to confirm the reliability of the ethnozoological information/data. Therefore, responses of information that were not in harmony with the earlier view were rejected, because they were considered as unreliable information/data. Only responses of an informant that were in harmony with those made earlier were taken as relevant and used for the data analysis.

### Specimen collection and identification

At the end of each interview, sample specimens of the animals cited for their medicinal use were collected, numbered and dried for identification. However, regarding animal species which were difficult to collect only their photographs were taken. The local names and associated attributes of the medicinal animals were recorded for each animal species. Preliminary identification was done in the field. The animal specimens which could not be identified in the field were taken to Arba Minch University (AMU). The medicinal animals were identified at AMU through visual comparisons with photographs and illustrations available on the internet and taxonomic keys of the fauna of Ethiopia.

### Data analysis

For the ethnozoological analyses the data were qualitatively and quantitatively assessed, using indices such as FL, ICF, and RFC values developed by Leonti [25] to determine the relevance of the collected data. The latter were processed with SPSS version 20 statistical software. Descriptive statistics were employed to categorize and summarize the data on the kinds of medicinal animals, the parts/products used to prepare medicinal potions and on the way the medicines were administered. The percentage of informants claiming use of a certain animal species for the same major purpose or ailment to treat was calculated by using the Fidelity Level (FL).

$${\text{Fidelity}}\;{\text{Level}}\;\left( {{\text{FL}}} \right)\% = \frac{{N_{{\text{p}}} }}{N}100$$

where *N*_p_ = The number of respondents that claim use of a species to treat a particular ailment. *N* = The number of respondents that use the animals as a medicinal agent to treat any given ailment.

The FL ranges from 1 to 100% (high values indicate that this particular animal species is used by large number of people; whereas, a low value shows that respondents disagree on the usefulness of a species in treating ailments).

The Informants’ Consensus Factor (ICF) was calculated for each category to identify the agreements of the informants on reported cures for the group of ailments. The ICF was calculated as follow;

$${\text{ICF}} = \frac{{{\text{Nur}} - N_{{\text{t}}} }}{{{\text{Nur}} - 1}}$$

where ICF = Informants Consensus Factor; Nur = Number of use citation in each category; *N*_t_ = Number of species used for a particular use category by all informants.

Low ICF values (near 0) mean that animals are chosen randomly or that there is no exchange of information about their use among informants; values approaching 1 mean that there is a well-defined selection criterion in the community or information is exchanged among informants. The ICF is used to calculate the degree of socio-cultural coherence regarding animals being used within and among certain communities with respect to similar ailments. The method rests on the assumption that the greater the degree of group consensus regarding the use of ethno medicinal species for treating certain conditions are, the greater the probability that the specific treatment is physiologically active or effective [24].

The Relative frequency of citation (RFC) index shows the local importance of each species. The RFC value was calculated using the formula

$${\text{RFC}} = \frac{{{\text{FC}}}}{N}$$

where FC = is the number of informants mentioning the use of a particular species and *N* = is the number of informants participating in the survey. The RFC index varies from 0 to 1. An RFC index of 0, means that nobody refers to the animal as useful, but an RFC index 1 indicates that all informants in the survey agreed that this animal is useful.

## Results and discussion

### Socio-demographic characteristics of the respondents

Data on the socio-demographic attributes of the informants with respect to age, residence, sex, marital and educational status, as well as occupation are given in Table 4. During the field survey, 132 individuals (112 men and 20 women) were interviewed. Male respondents were in the majority, because most of the traditional medicinal treatment in the Kucha area was meted out by male practitioners. This kind of male dominance regarding traditional medicinal uses has also been reported from northern Ethiopia where mostly males took part in interviews, in questionnaires and group discussion [20]. Since 98% of the respondents were of the Gamo ethnic group, the conclusions are therefore based almost entirely on the views of the Gamo people.

**Table 4:** Socio-demographic characteristics of the informants

Characteristics	Variables	Frequency	Percentage
Sex	Male	112	84.8
	Female	20	15.2
Residence	Rural	109	82
	Urban	23	17.4
Age (years)	25–34	6	4.5
	35–44	12	9
	45–54	32	24.2
	55–64	54	40.9
	≥ 65	28	21.2
Marital status	Single	4	3.0
	Married	123	93.2
	Divorced	2	1.5
	Widowed	3	2.3
Educational level	Unable to read and write	75	56.8
	Read and write	53	40.2
	College diploma and above	4	3.0
Occupation	Farmer	56	42.4
	Merchant	9	6.8
	Private traditional health service	15	11.4
	Farmer & private traditional health service	40	30.3
	Merchant & private traditional health service	12	9.1

The majority of the informants (*n* = 54, 40.9%) were within the age range of 55–64; while, 32 (24.2%) were 45–54 years old and 12 (9%) were 35–44 years old. The majority of the interviewees lacked formal schooling due to the unavailability of modern education and the remoteness of educational institutions as well as fallacious traditional personal attitudes to sub-districts modern education. Of the total respondents, 75 were illiterate, and the remaining 57 had attended primary school and above. Most of the respondents (42.4%) were farmers who had a thorough understanding of ethno-medicine and 30.3% of them provided private traditional health services (Table 4). Almost all the informants were neither officially registered nor organized within the modern health service delivery systems in their communities.

### Medicinal animals and animal parts/products used to treat human ailments

People of the study area collect different animal parts for the preparation of traditional drugs. Animals and their parts/products were found to be used for the treatment of around 35 different kinds of ailments including malaria, headache, rabies, anaemia, and cough. The animals were used either whole or provided parts or certain products, e.g., milk, blood, meat, teeth, honey, etc., (Table 5), to be used in treating a variety of ailments. With regard to the animal parts/products used for medicinal purposes, honey, milk, and butter were the most widely used products in the traditional medicinal armamentarium (26.9%), followed by meat and fat (21.2%). Whole body animal and external body parts were used in 9.6% of the cases and visceral organs such as liver, tongue, gastric content and bile were used in 7.7% of all cases. Excreta (stool and urine), bones and teeth as well as blood scored an identical acceptance level of 5.8%; while, larvae (3.8%) and saliva as well as sweat (3.8%) were the least favoured medicinal material (Table 5).

**Table 5:** Animal parts or products used as traditional medicine in the study area (52 in total)

Medicinal parts/products of animals	No. of parts/products used	Percentage (%)
Products (honey, milk, butter)	14	26.9
Meat and fat	11	21.2
Whole body	5	9.6
External body parts (skin, eyelashes, hair, fur)	5	9.6
Visceral organs (liver, tongue, gastric content, bile, etc.	4	7.7
Bone and teeth	3	5.8
Blood	3	5.8
Excreta (stool and urine)	3	5.8
Larvae	2	3.8
Saliva and sweat	2	3.8

Findings from Ethiopia [5, 13, 15], as well as from Brazil [3, 8], and India [9–11, 14] had revealed that the two animal products most commonly used in addition to the aforementioned animal parts, were meat and fat (18%–27.5%, respectively). In the Wolayta district of southern Ethiopia 21.1% of the animal-based medicinal remedies were prepared from animal products such as honey, milk, butter, cheese, and eggs [25], but for the inhabitants of Assam in India it could be shown that the use of whole animals in treating ailments was with 44.9% the major method, followed by using what was then referred to by the authors as “animals parts such as meat” (22.5%). The differences between the results obtained by different investigators could, of course, reflect different healing approaches, but it could also be a consequence of dissimilar interpretations or definitions what constitutes an animal part and what is considered an animal product.

### Indigenous knowledge with regard to preparation method

The local community employed various methods of preparation of traditional medicines for different types of ailments (Table 6). Direct use as with the consumption of raw or fresh material, drying, powdering and mixing with other ingredients, preparing soups and stews, were some of the most common preparation methods used in treating humans suffering from a medical condition. The principal methods for the preparation of the animal parts or products included in a remedy were, apart from the direct raw or fresh use of the material (50%), drying (11.5%) and cooking a soup or a strew (7.7%). Investigators from different parts of the world had also reported that the most popular methods in their studies on the preparation of a traditional remedy to fight a disorder had also been based on the consumption of some fresh material of the animal [5, 13–15, 27]. Therefore it seems that the raw consumption of animals or animal parts as part of the therapeutic process is a common practice among various ethnic communities worldwide [4, 13, 16, 21, 28].

**Table 6:** Methods used in preparing animal-based medicines (53 in total)

Types of preparation	No. of preparation	Percentage (%)
Direct use of raw and/or /fresh animal material	26	49.1
Drying	6	11.3
Cooking	4	7.6
Preparing a soup and/or a stew	4	7.6
Drying and powdering	3	5.6
Drying, powdering and mixing other ingredients	3	5.6
Warming or melting	3	5.6
Drying and smoking/burning (e.g., fumigation)	2	3.8
Fresh/raw material mixed with other ingredients	2	3.8

### Indigenous knowledge on the mode of application

This study showed that modes of application/administration of the traditional medicines varied depending on the part/product of the animal used and the type of the ailment condition to be treated (Table 7). The routes of administration of these medications are eating, drinking, anointing, attaching, dropping, holding, exposure to fumigation, banding and inhalation. For instance, both solid and liquid remedies were administered orally if applicable, otherwise they were applied to the skin by tying, rubbing, massaging, anointing, and fumigation to allow the potent components of the medicine to enter the body. In the study area, oral (54.2%) is the dominant route of administration followed by the dermal (39.6%) application. This result is in line with various studies in Ethiopia and other countries [5, 14–17, 27]. The authors of these papers all reported that the major way of administration, i.e., 33.8–88.1% (depending on area and tribe), was oral ingestion. On the other hand, that conclusion is not in agreement with a recent survey from the Arba Minch Gamo Zone in Ethiopia that showed that the highest route of administration was dermal, accounting for 50 percent [13].

**Table 7:** Major methods (routes) of traditional medicine administration and delivery (48 in total)

Mode of applications	No. of application	Percentage (%)	Mode of delivery
Eating	14	29.2	Oral
Drinking	12	25.0	Oral
Anointing	7	14.6	Topical/dermal
Tying	5	10.4	Topical/dermal
Dropping	4	8.2	Topical/dermal
Holding	3	6.3	Topical/dermal
Fumigation	1	2.1	Nasal
Banding	1	2.1	Topical/dermal
Inhalation	1	2.1	Nasal

### Views on how to share and transfer knowledge on medicinal animals

The local community exploits their shared traditional knowledge to manage health problems at home by using different animals and their parts/products found around them before looking for other options regardless of the type of health problem and its intensity. According to the informants of the study area, their preference for traditional medicine was because of the lack of substitutes for some of the diseases in the modern healthcare services. For example, diseases believed to be caused by the urine of a bat, not only are easy to access but also cost little.

The majority of the informants 36 (27.3%) obtained their knowledge on the use of the medicinal animals and their parts/products from their father (Table 7). About 98 (74.2%) of the informants were interested to transfer their medicinal knowledge to the next generation. Similarly, it was reported that most of the people of Motta City’s administration and the Hulet Eju Enessie District were interested to transfer their medicinal knowledge to the next generation [5]. Most of the informants 128 (96.9%) stated that traditional medicinal services were accepted by the local community. This indicated that conventional medicines are still considered useful and important, especially for the poor who have little access to modern medicines and do not have the money to pay for expensive drugs and often do not trust them. A rather similar conclusion was reached for other Ethiopian [5, 13, 15, 27] and Tanzanian tribals in Africa [29] (Table 8).

**Table 8:** Indigenous knowledge transfer practice

Indigenous knowledge	Variable	Frequency	Percentage
Source of knowledge	Father	36	27.3
	Mother	18	13.6
	Grandfather	29	21.9
	Friends	21	15.9
	Trial and error	1	0.8
	Others (oral tradition)	27	20.5
Acceptability of the traditional medicine Service by the community	Acceptable	128	97.0
	Not acceptable	4	3.0
Interest to transfer the medicinal knowledge to the next generation	Interested	98	74.2
	Not interested	34	25.8
The benefit obtained from traditional medicinal service	Income source	130	98.5
	Free service/satisfaction	2	1.5

### Medicinal animal of the region: species diversity

The present study revealed a wealth of knowledge on the use of traditional animal-based medicines in treating many types of ailments by the inhabitants of Kucha District, Southern Ethiopia. A total of 24 medicinal animal species were recognized by the local community. Regarding the classes of the medicinal animals, mammals were represented by the highest number of species (13 = 54.2%), followed by birds with five reported species (20.8%). Arthropods were represented with four species (16.7%) and reptiles and fish contained one species each (4.2%) (Table 9). Amphibians and invertebrates other than arthropods were not mentioned. This result agrees with the information obtained from FGD participants and findings from several studies in Ethiopia and other countries, which also reported mammals with 12–27 species being the dominant source of medicinal animals [5, 8–13, 27–29]. This outcome demonstrates that the interviewed people of the Kucha district have therapeutic uses for only a relatively small number but taxonomically rather wide range of diverse species of animals to treat diseases and bodily malfunctions. The findings indicated that the local people over a wide area in Ethiopia show a tendency to use the same medicinal animals and this can be an indicator of the genuine therapeutic value of these animals as well as the sharing of the indigenous knowledge on therapies.

**Table 9:** Major animal groups and number of species used in the traditional medicine (24 in total)

Animal groups	Number of species	Percentage (%)
Mammals	13	54.2
Birds	5	20.8
Arthropods	4	16.7
Reptiles	1	4.2
Fish	1	4.2

### Assessing a medicinal animal’s condition

The local healers of the study area employ several criteria to assess an animal’s condition (Table 10). Thirty eight (73.1%) preparations are made from fresh material, followed by dry 10 (19.2%) and both dry and fresh condition 4 (7.7%). Findings that were previously reported from various areas of Ethiopia and several other countries also indicated that 39.9%-56.6% of the medicinal animals were used in their fresh state [5, 13, 15, 20] but for the tribals of the Pachamalai hills of Tamil Nadu, India [28] and the Sukuma tribe of the Busega district in North-western Tanzania it has been reported that the dry condition (47.83%) was dominant [29]. This difference in the medicinal animals’ condition may be linked to differences in the socio-cultural beliefs that the animals are held in and the practices of the healers in the various tribal regions.

**Table 10:** Preferred condition of the medicinal animal and/or animal product

Medicinal animals condition	No. of medicinal animals condition	Percentage (%)
Fresh	38	73.1
Dry	10	19.2
Both fresh and dry	4	7.7

### Traditional medicinal animal preservation methods and use of additives

According to ten knowledgeable healers (key informants) the five most common preservation methods are: wrapping in cloth sheet, hanging under the roof, storing in a plastic bag, keeping the material in a clay container, and storage in sealed bottles. The use of cloth sheet was ranked as number one by traditional practitioners for the preservation of medicinal animals followed by roof hanging, plastic bags, clay containers, and sealed bottles (Table 11). According to the discussions with the healers, the preparations were made from mixtures of different animal species with different substances added such as water, butter, honey, oil with spices and garlic. However, there were cases in which such additives were not used. The extra substances have a double function, e.g., to improve the flavor of a potion and to reduce adverse effects such as vomiting and diarrhea, and to enhance the efficacy and healing conditions, as reported by focus group discussion participants.

**Table 11:** Ranking by 10 key informants of the preferred preservation method of the medicinal animal and/or animal part (use values: 4 = best, 3 = very good, 2 = good and 1 = less used)

Preservation methods	*R*_1_	*R*_2_	*R*_3_	*R*_4_	*R*_5_	*R*_6_	*R*_7_	*R*_8_	*R*_9_	*R*_10_	Total	Rank
Cloth sheet	4	4	4	3	4	4	4	4	4	4	39	1st
Roof hanging	4	4	3	4	3	4	4	3	3	2	34	2nd
Plastic bags	3	2	3	3	4	3	4	4	3	2	31	3rd
Clay container	1	1	2	1	1	2	1	2	1	1	13	4th
Sealed bottles	1	2	1	1	1	1	1	1	1	1	11	5th

### Medicinal animal popularity in the community and bio-cultural value of a species

**Fig. 5:**
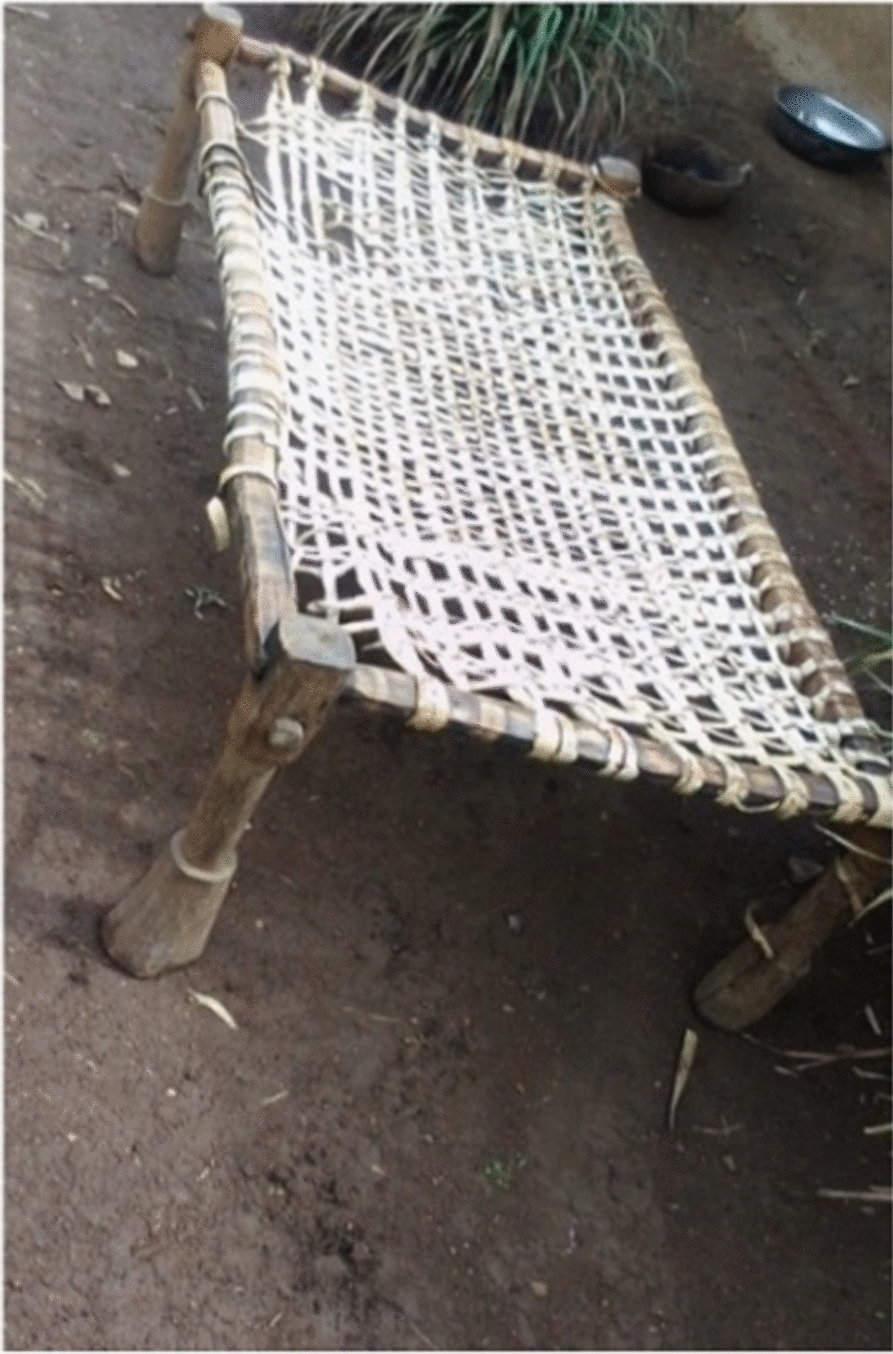
Bedposts made of bone

**Fig. 6:**
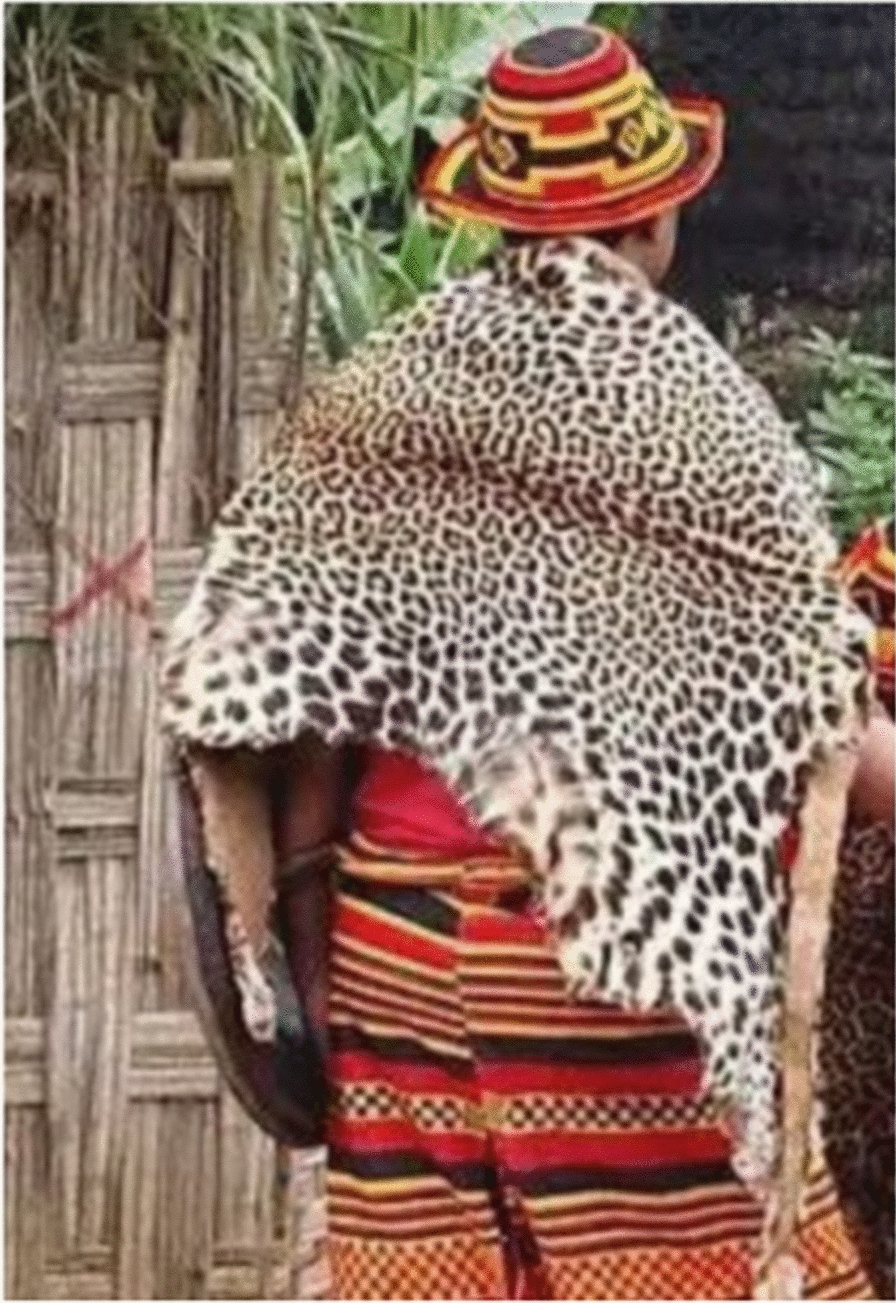
Animal fur as traditional cape

**Fig. 7:**
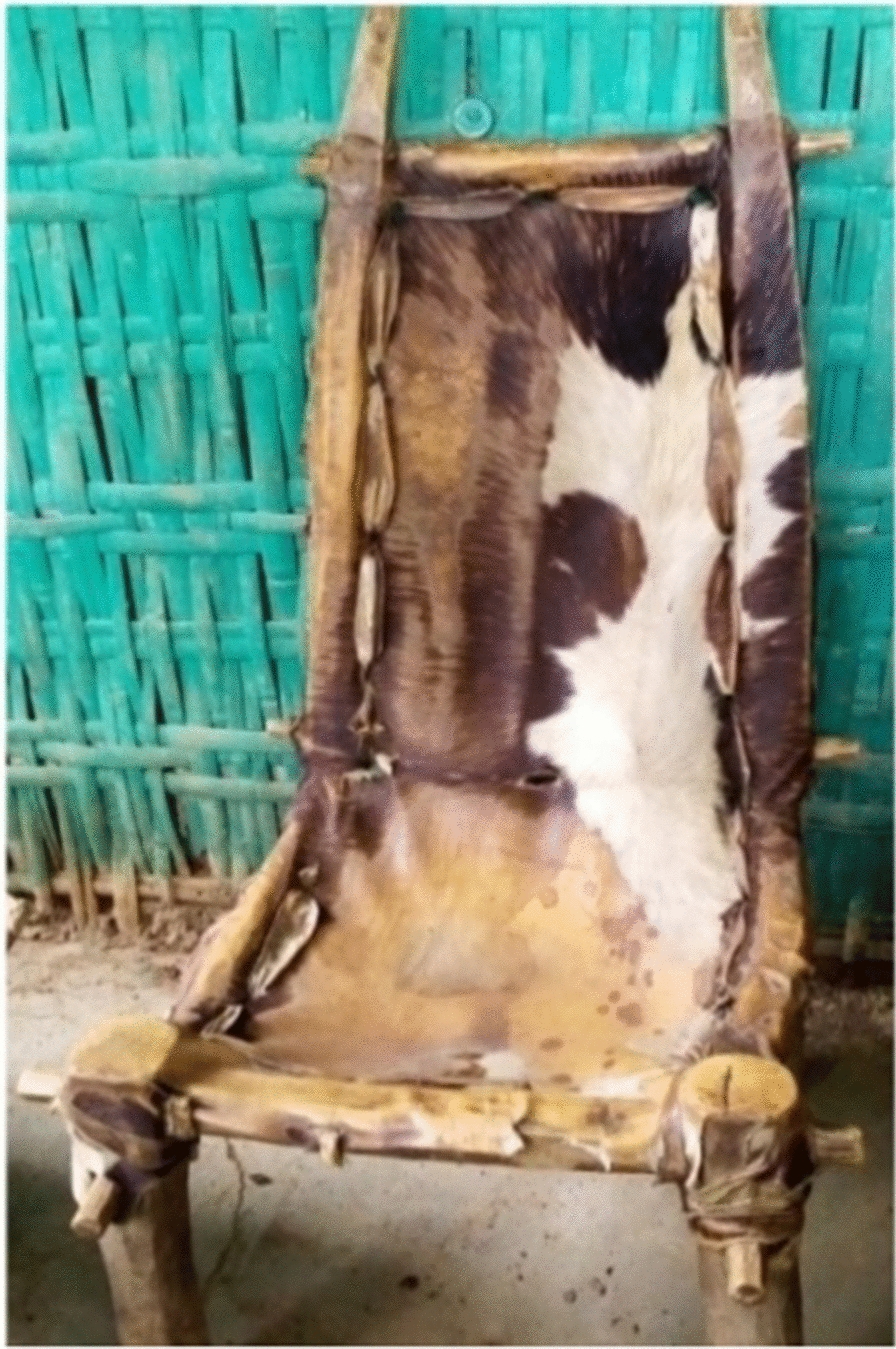
Animal skin as part of a chair

**Fig. 8:**
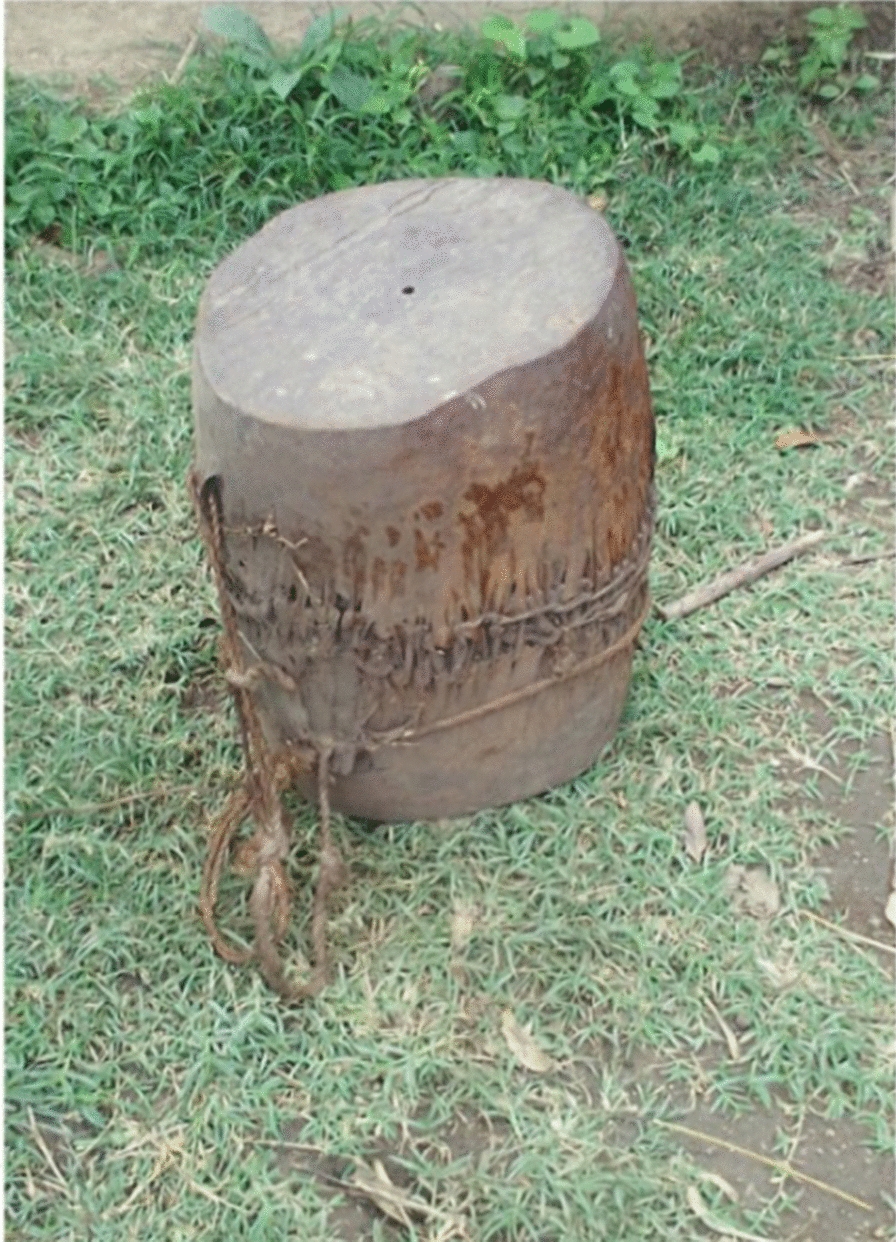
Drum of animal hide

**Fig. 9:**
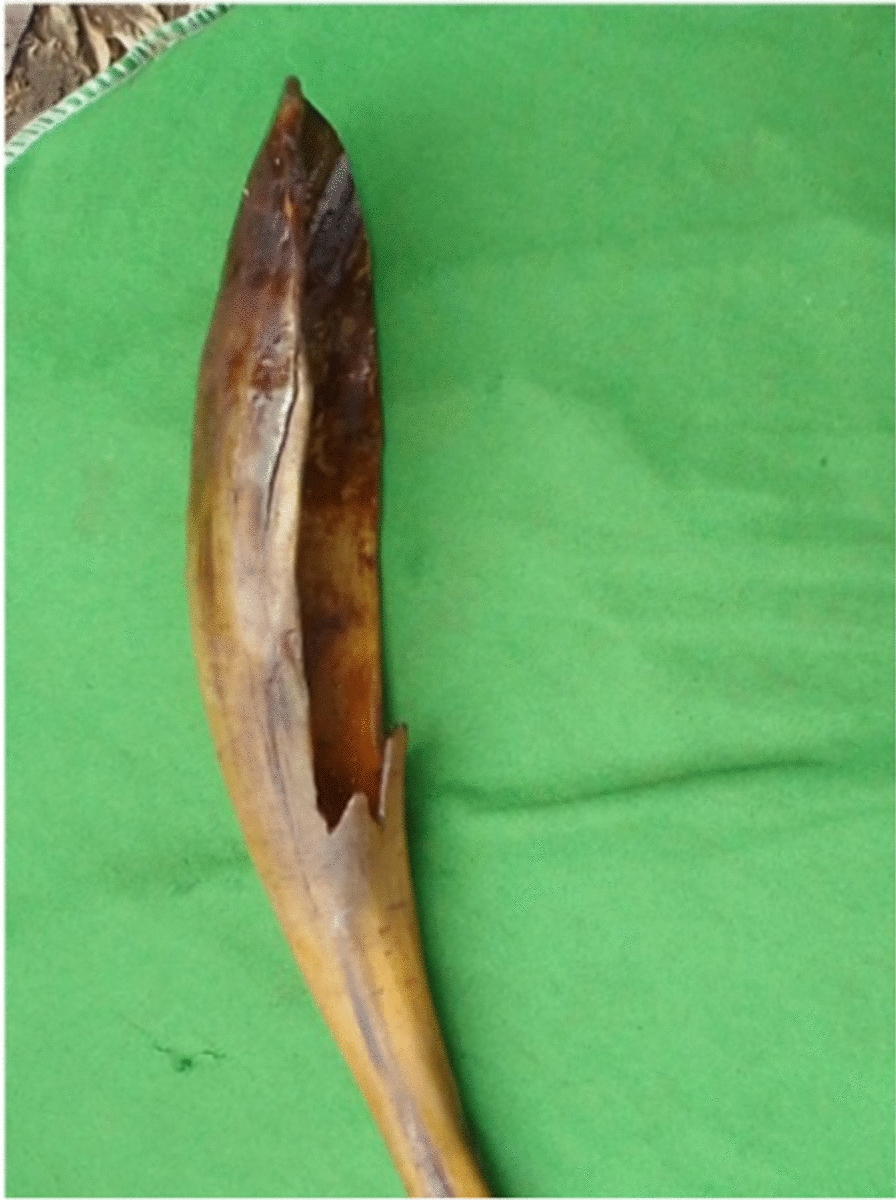
Bone spoon for porridge (*mokae*)

**Fig. 10:**
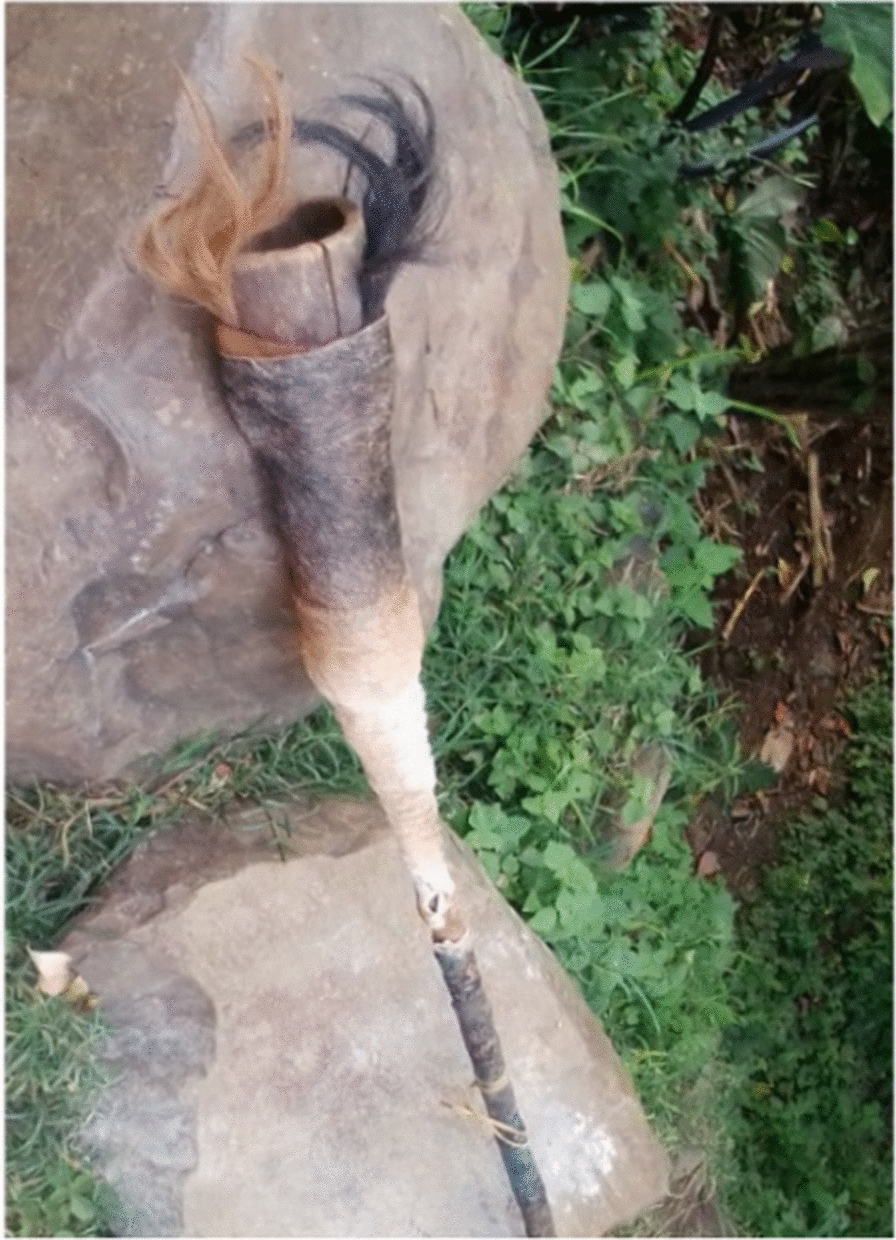
Musical instrument made of animal horn

**Fig. 11:**
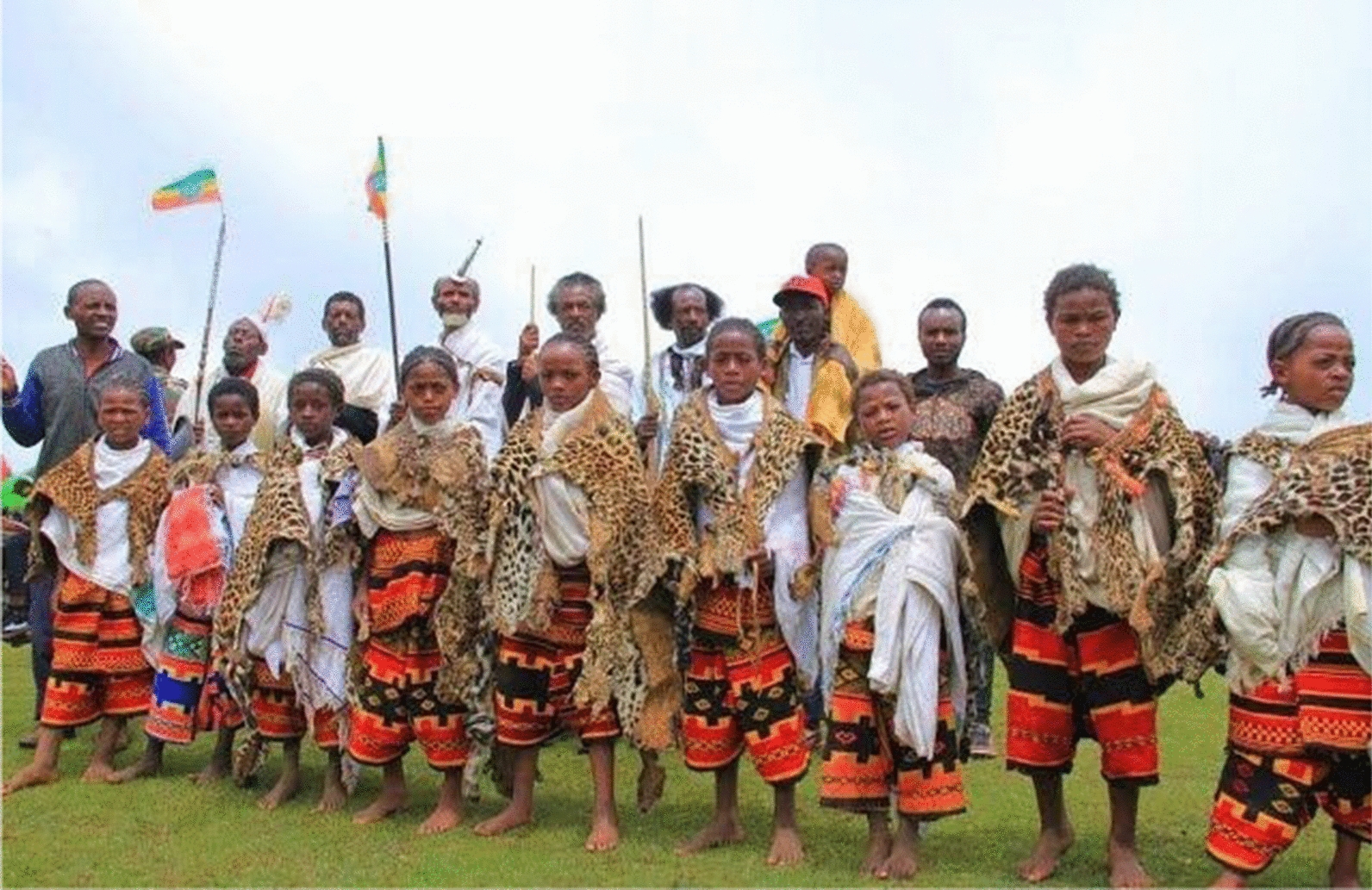
Various animal products are used as decorations and auxiliaries by the Kucha people during celebrations

Faunal resources have played a wide range of roles in the study area. Other than for medicinal purposes, Kucha people also used animal resources for various other aspects in their daily life. The Kucha people use slough (molted skin of various animals) or animal skins for clothes worn during mourning and celebrations, for making drums and protecting musical instruments or tableware, etc. (Figs. 5, 6, 7, 8, 9, 10, 11). Same animals or their parts may be used to decorate traditional houses, which once again are uses that have also been reported from other tribes in Ethiopia as well as other countries [3, 29]. Similarly, it has been documented that various tribal groups sacrifice animals in connection with a variety of rituals and that they may be turned into a variety of traditional tools [1, 14, 15, 17].

### Relative frequency of citation

The Relative frequency of citation (RFC) index was calculated to determine the local importance of each species (Table 12). The most cited animal species were: the *cow* (RFC = 1.0), the *chicken* (RFC = 0.7), the *human*, the g*oat* and different *sweat bee* species with an RFC = 0.6, *honey bee* (RFC = 0.5) *porcupine* and *hyena* with an RFC = 0.3, and domestic *cat* and *fishes* with an RFC = 0.2). The *leopard* scored the lowest value (RFC = 0.1); while, the highest RFC index of the cows demonstrates the importance of this species as a source of medicines. However, if animal species scored low RFC values like, for instance, the leopard, this does not necessarily mean that they are not important locally. It may be that most of the respondents were not aware of the species’ therapeutic potential or that the species was so rare that it was rarely used. However, vertebrates do appear to be the main source of animal-derived medicines not just in Ethiopia [5, 13, 22] but also in South America [2, 3, 8], Africa [4, 26, 29], India [9–11, 30] and even Europe [7].

**Table 12:** Relative frequency of citation for the top 10 animals

Animal species	Number of informants mentioning about the use of the species (FC)	Number of informants participating in the survey (*N*)	Relative frequency of citation (RFC)
Cow (*Bos taurus*)	130	132	1.0
Chicken (*Gallus domesticus*)	87	132	0.7
Goat (*Capra aegagrus*)	82	132	0.6
Human (*Homo sapiens*)	76	132	0.6
Sweat bee (*Halictus scabiosae*)	72	132	0.6
Honeybee (*Apis mellifera*)	68	132	0.5
Hyena (*Crocuta crocuta*)	42	132	0.3
Porcupine (*Hystrix cristata*)	38	132	0.3
Cat (*Felis domesticus*)	32	132	0.2
Leopard (*Panthera pardus*)	22	132	0.1

### Informant consensus factor

Malaria, children’s eye diseases, wounds, breast pain, swelling glands, tetanus and cough ailment categories had the highest informant consensus factor values (ICF = 1), followed by cold and bone fractures with an ICF of 0.9. When only one animal species or a few were said to be employed by a large number of informants, the ICF value is high. However, headache (ICF = 0.8) had a lower ICF value than the other categories. Thus, a low ICF value suggests that informants use that particular animal rarely to treat diseases or disorders. Several different animal species were used in connection with headache (ICF = 0.8**)** (Table 13).

**Table 13:** Informant consensus factors for 10 common indications for medicinal animal and animal product

Indication	Number of use reports (Nur)	Number of species for the indication (Ns)	Informant consensus factor (ICF)
Malaria	15	1	1
Children eye disease	22	1	1
Wound	24	1	1
Breast pain	21	1	1
Swelling glands	32	1	1
Tetanus	24	1	1
Cough	53	3	1
Cold	97	5	0.9
Bone fracture	26	3	0.9
Headache	27	3	0.8

### Fidelity level of a medicinal animal

Fidelity levels (FL) demonstrate the percentage of respondents that agree on the validity of a certain animal or its product to cure a sick individual of an illness. The fidelity level in our sample varied from 1.5% to 98% on the basis of respondents claiming the use of certain animals for the same purpose. Cows, for example, to treat humans suffering from worms, headache, earache, common cold, bone fracture and weight gain scored the highest FL value (*n* = 130; 98%) followed by chicken egg yolk as a remedy for cough and body weight gain (FL: *n* = 87; 65.9%), goats to treat common colds, blood for anaemia and meat for eight gain (*n* = 82; 62%) at morning and evening. Human breast milk was reported to ameliorate eye conditions in children, urine to treat wounds, hair and saliva to ward off the “evil eye” (FL: *n* = 76; 57.6%); The larvae of sweat bee species were used to treat coughs (fresh larvae) and tetanus (cooking fresh larvae and mixed with honey and butter) (FL: *n* = 72; 54.5%); while, honey bees through anointing, eating and holding them were involved in treating cleft lip, erectile problems, cold, physical appearance, and swellings (FL: *n* = 68; 51.5%). To treat sleep disorders eyelashes of the hyena tied to the neck of the sufferer were mentioned, while the hyena’s teeth and tongue tied to the neck were thought to be able to ward off the “evil eye” (FL: *n* = 42; 31.8%), Cooking fresh meat of porcupine species mixed with oil and spices were used in cases of colds, rheumatism, pleurisy and asthma (FL: *n* = 38; 28.8%). Tying the dry and powdered bones of cats were sought to cure swelling glands (FL: *n* = 32; 24.2%); while, inhalation sweat and eating the dry meat stew of a leopard (used to treat headache and rabies) with just 22.16% had the lowest fidelity level value (Table 14). The results indicate that in many cases the same animal species were reported to be used for the treatment of more than one ailment. This trend has also been found in different regions of the world [3, 5, 27–31] and was explained by Meyer-Rochow to be based, firstly, on the fact that different parts or organs of the same animal were used and, secondly, that preparation methods differed for different ailments [32]. On the other hand, different animal species were sometimes used to treat an identical disease, which was also explained by Meyer-Rochow on the basis that animal species, no matter how different in looks and appearance, all possessed organs common to all of them, for example, heart, blood, skin, brain, liver, bile, etc., and that the species most readily available locally would be the preferred source [32].

**Table 14:** Fidelity level for the top 10 medicinal animal species used in treating diseases in the study area (for scientific names of species, see Table 12)

Animal species	Indication	Number of informants for the indication	Total number of informants participating in the survey	FL (%)
Cow	Anaemia, malaria, common cold, headache, toxin, bone fracture, problem with feet, worms, earache, weight gain, cough, pleurisy, cold, abdominal pain	130	132	98%
Hen	Cough, cold, abdominal pain, pleurisy, fever, common cold, weight gain, and swelling wounds	87	132	65.9%
Goat	Anaemia, common cold, fever, and weight gain	82	132	62%
Human	Children’s eye disease, wound, and evil eye	76	132	57%
Sweat bee	Cough, tetanus, asthma, breathing, rheumatism	72	132	54.5%
Honey bee	Cleft lip, erectile problem, cold, beauty of skin and swelling wound	68	132	51.5%
Hyena	Oversleeping, evil eye	42	132	31.8%
Porcupine	Cold, rheumatism, pleurisy and asthma	38	132	28.8
Cat	Swelling glands	32	132	24.2
Leopard	Headache, rabies virus	22	132	16.7

### Major threats to medicinal animals

According to our ten key informants, the major threats to medicinal animals in the study area (Table 15) were habitat loss (97.5%), overexploitation of the animal resource (95%), climate change (87.5), invasive species (62.5%) and pollution (30%). In agreement with this finding, it had also earlier been reported that habitat conversion (25%-60%) and overexploitation (34%–45%) were seen as the main factors for the disappearance of the medicinal animals in Ethiopia [13, 27, 29]. This is in agreement with the observation that medicinal animals can become heavily affected by habitat losses and decreases in the native vegetation due to agricultural expansion, deforestation, overgrazing, and fuel wood collecting [3, 17, 33].

**Table 15:** Priority ranking by 10 respondents of 5 factors perceived as threats to medicinal animals (use values: 4 highest, 3 = high, 2 = moderate and 1 = less important)

Threatening factors	*R*_1_	*R*_2_	*R*_3_	*R*_4_	*R*_5_	*R*_6_	*R*_7_	*R*_8_	*R*_9_	*R*_10_	Total	Rank
Habitat loss	4	4	4	3	4	4	4	4	4	4	39	1st
Overexploitation	4	4	3	4	4	3	4	4	4	4	38	2nd
Climate change	3	4	4	3	4	2	4	3	4	4	35	3th
Invasive species	2	3	3	4	1	2	4	3	1	2	25	4th
Pollution	2	1	2	1	1	1	1	1	1	1	12	5th

### Conservation practices in relation to the medicinally important species of the study area

The effects caused by humans on the natural habitat of medicinal animals are serious problems for the conservation of these animals [13, 17] and also, given the still widely practiced traditional treatments of diseases involving animals and their parts, for the health of the inhabitants of the Kucha District. Conservation practices put in place by concerned bodies and the local residents were, regrettably, largely ineffective and most species considered medicinally valuable, are becoming increasingly less common. The majority of the informants (84.8%) revealed that there are no or only limited conservation activities in the study area to manage those animal species deemed important as sources for traditional medicinal treatments. However, the remainder of the informants (15.2%) stated that there are a number of medium and high conservation and management practices in the study area. Evidence indicates that the local community’s knowledge of the use of the animal resource is very important for conservation efforts and therefore needs to be paid attention to and acted upon by administrators [27, 33].

## Conclusion and outlook

Our study of the medicinal animals and their products used by inhabitants of the Kucha district revealed that the community commonly uses a variety of animals for primary healthcare, as summarized in Table 16. Traditional medicinal practices continue to be of considerable importance to this Ethiopian community, especially in view of illnesses and afflictions that are often encountered such anaemia, headaches, common cold, pleurisy, skin problems, attacks by worms, etc., and for which various folk medicinal treatments exist. For the locals traditional medicines are usually still cheaper and more readily obtainable than doctor-prescribed drugs and potions that need to be ordered from afar, paid for with much money and be administered by so-called experts that are not always trusted by the locals. The local people still prefer to put their trust in methods that have stood the test of time and been followed in their area for hundreds of years, rather than rely on medicines that they would see as ‘unproven’. To convince the population that traditional and modern medicine can complement each other is an educational problem that needs to be resolved.

**Table 16:** Medicinal animals used in the treatment of diseases: scientific name, local name, habitat, parts/products used, method of preparation, type of administration in connection with the disease or illness to be treated

Animal group	Common English Name	Local name Gammotho (G) Amharic (A)	Scientific name	Animal habitat	Parts/products used and preparation method	No. of parts or product used	Disease (s) treated	No. of ills treated	Administration method
Mammal	Wild Goat	Deeshshaa(G) Fiyel(A)	*Capra aegagrus hircus* L	Wild and Domestic	Fresh whole animal mixed with butter, oil and spices to prepare a tasty soup	1	Common cold	1	Drinking
					Fresh blood, milk	2	Anaemia, fever	2	Drinking
					Fresh meat of animal mixed with Butter, oil and spices to prepare roast	1	Weight gain	1	Eating
Mammal	Cattle (domestic cow)	Mizza(G) Lam(A)	*Bos taurus*	Domestic	Fresh liver	1	Anaemia	1	Eating
					Fresh bile	1	Malaria	1	Drinking
					Fresh/Dry fat	1	Cracking the heel	1	Anointing
					Melting the fresh butter of a black cow	1	Worms	1	Drinking
					Fresh butter directly	1	Headache	1	Anointing
					Melting Fresh butter	1	Headache	1	Nasal Drops
					Melting fresh butter	1	Ear pain	1	Drops into ear
					Fresh butter mixing with Garlic & red meat (Gugo)	1	Cold and Bone fracture	2	Eating
					Fresh butter mixed with garlic and honey	1	Cough, pleurisy, Common cold	3	Drinking
					Fresh butter mixed with different foods	1	Bone fractures, weight gain	2	Eating
					Fresh milk	1	Toxin, weakness, teeth problems, Abdominal pain, weight gain	4	Drinking
Mammal	Bat	Wurkawurko(G) Yelelit wof(A)	*Cynopterus sphinx*	Wild	Fresh blood and meat directly	2	Skin disease	1	Anointing
Mammal	Porcupine	Quxarssa(G) Jart(A)	*Hystrix cristata*	Wild	Cooking fresh meat mixed with oil and spices	1	Common cold, rheumatism, Pleurisy, asthma	4	Eating
Mammal	Bush pig	Guduntoa(G) Asama(A)	*Potamochoerus larvatus*	Wild	Cooking fresh meat	1	Hepatitis, fever	2	Eating
Mammal	Dog	Kanaa(G) Wusha(A)	*Canis familiaris*	Domestic	Fur is burnt	1	Rabies virus	1	Ash put on bite
Mammal	Human	Asaa(G) Sew(A)	*Homo sapiens*	Domestic	Fresh breast milk	1	Children’s eye disease	1	Dropping
					Fresh urine	1	Wound	1	Drops
					Fresh hair taken from Evil man and warped in cloth	1	Evil eye	1	Tying on neck
					Fresh Evil man’s saliva Directly	1	Evil eye	1	Holding
Mammal	Domestic cat	Gawaraa(G) Dimet(A)	*Felis domesticus*	Domestic	Drying and powdering bone warped in cloth	1	Swelling of glands	1	Tying on neck
Mammal	Warthog	Gaashuwa(G) Kerkero(A)	*Phacochoerus africanus*	Wild	Heating the dried teeth	1	Breast pain	1	Fumigation
Mammal	Hyena	Godaree(G) Jib(A)	*Crocuta crocuta*	Wild	Dry eyelash wrapped in cloth	1	Oversleep	1	Tying to the neck
					Dry tongue and teeth crushed and wrapped in cloth	2	Evil eye	1	Tying to the neck
Mammal	Baboon	Geleshshuwa(G) Zinjero(A)	*Papio anubis*	Wild	Dried or fresh feces mixed with water	1	Evil eye	1	Drinking
Mammal	Leopard	Zerussa (G) Aboshemane(A)	*Panthera pardus*	Wild	Fresh sweat removed from the leopard when it touches the wood as it passes	1	Headache	1	Inhalation (sweat)
					Dried meat directly, Preparing stew from the Meat	2	Rabies virus	1	Eating
Mammal	Common duiker	Geneaa(G) Midakuwa(A)	*Sylvicapra grimmia*	Wild	Fresh gastric content (Fers) directly	1	Donkey disease (Gande)	1	Eating
Bird	Pigeon	Haraphphea (G) Rigib (A)	*Columbiformes spp*	Wild	Fresh egg directly	1	Pleurisy	1	Drinking
Bird	Helmeted Guinea Fowl	Suckulo(G) Jigra(A)	*Numida meleagris*	Wild	Cooked meat	1	Cold	1	Eating
Bird	Chicken	Kuto(G) Doro(A)	*Gallus gallus domestics*	Domestic	Fresh egg yolk directly	1	Cough, common cold, Abdominal pain, pleurisy, fever, common cold	5	Drinking
					Cooking whole body mixed with ingredients	1	Weight gain	1	Eating
					Melting fatty meat and mixed with ash	1	Swollen wound,	1	Anointing
Bird	Eagle	Golle(G) Nisire (A)	*Haliaeetus* sp.	Wild	Dry feces mixed with water	1	Evil eye in livestock	1	Drinking
Bird	Raven	Qooraasiya (G) Kura (A)	*Corvus corax*	Wild	Fresh egg directly	1	Asthma	1	Drinking
Arthropoda	Honey bee	Matta(G) Nib(A)	*Apis mellifera*	Domestic	Fresh honey directly	1	Cleft lip	1	Anointing
					Fresh honey direct	1	Erectile problem, common cold; Beauty of the skin	3	Eating
					Whole animal by letting the bee sting	1	Wound swelling	1	Holding
Arthropoda	Tick	Danqquwa(G) Mezhiger(A)	All tick species	Wild	Fresh blood directly	1	Skin disease	1	Anointing
Arthropoda	Scorpion	Masimasuwo(G) Gint (A)	*Palamnaeus* *swammerdami*	Wild	Fresh whole body directly	1	Scorpion toxins (Skin disease)	1	Anointing
Arthropoda	Sweat bee	Degraa essa(G) Tasma nib(A)	*Halictus scabiosae*	Wild	Fresh larvae directly	1	Cough, asthma, rheumatism	3	Eating
					Cooking fresh larvae, mixed with honey and butter	1	Tetanus	1	Eating
Fish	Fish	Molle(G) Assa(A)	All fish species	Wild	Whole body prepared as a soup	1	Cold	1	Drinking
					Cooking meat mixed with ingredients	1	Bone fracture	1	Eating
Reptile	Snake	Shosha(G) Ibab(A)	*Naja naja*	Wild	Dry powder of the snake skin/scales	1	Urine problem	1	Banding on navel

Relevant knowledge is passed on via oral transmission, imitation and application. The community has been encouraged to show an awareness of the animals and their accessibility to treat illnesses and disorders. Of the 24 medicinal animal species that were identified, mammals and birds had the highest uses. Most of the medicinal animals (17 = 71%) were obtained from the wild; while, 7 (29%) were from domestic sources. Twenty-two species (91.7%) were used to treat human ailments in ten different categories of maladies and 2 (8.3%) were only used to treat livestock ailments. Altogether at least 20 different kinds of specific conditions (Table 16) were mentioned to benefit from traditional treatments administered by traditional healers.

Yet, relatively few species were used in connection with both human as well as animal complaints. Whole bodies or body parts and products of the therapeutic species were frequently involved in traditional treatments. It was obvious that most of the members of the local communities studied, knew that certain animals and/or their parts could be prepared and administered, often together with specific ingredients, to a sick individual. Honey, milk, butter or eggs were the most frequently used medicinal animal products, although ground bones, horns or antlers also had some uses. In spite of the importance of the traditional treatments, efforts to document, safeguard and manage this wealth of indigenous skills and knowledge have been minimal and important indigenous wisdom is in danger of getting lost together with the elders and the local experts. Hence, it is important to register and chronicle the various folk medicinal ways in which animals and their products can bring about an individual’s recuperation and convalescence.

## Data Availability

To be obtained from M.K. on request

## Abbreviations

AMU: Arba Minch University
FAO: Food and Agriculture Organisation
FC: Number of informants mentioning the use of a particular species
FGD: Focus group discussion
FL: Fidelity level
ICF: Informants’ Consensus Factor
KII: Key Informant Interview
*N*: Number
*N*_p_: Number of respondents agreeing on a species use for one ailment
*N*_t_: Number of species used for a particular disease category
Nur: Number of citations in each category
PA: Peasant Association
RFC: Relative Frequency of Intention
SNNPR: Southern Nations Nationalities and Peoples Region
USA: United States of America
WHO: World Health Organisation
WRI: World Resource Institute
